# THE EFFECT OF PICEATANNOL FROM PASSION FRUIT SEED EXTRACT ON SIRTUIN EXPRESSION

**DOI:** 10.1093/geroni/igae098.4085

**Published:** 2024-12-31

**Authors:** Motoki Tsukiashi, Shinpei Kawakami, Hiroko Maruki-Uchida, Naoki Iemoto

**Affiliations:** Morinaga & Co., Ltd., Yokohama, Kanagawa, Japan; Morinaga & Co., Ltd., Yokohama, Kanagawa, Japan; Morinaga & Co., Ltd., Yokohama, Kanagawa, Japan; Morinaga & Co., Ltd., Yokohama, Kanagawa, Japan

## Abstract

Piceatannol, a polyphenol abundantly found in passion fruit seeds, is structurally similar to resveratrol. It has four hydroxyl groups, is highly water-soluble, and is expected to have various effects, including antioxidant properties. Sirtuins are known to promote longevity and prevent various chronic diseases associated with aging. Given the growing interest in longevity research, this study aimed to explore the potential of piceatannol to induce sirtuins. We hypothesized that piceatannol might function as a more potent activator of sirtuins than resveratrol. Using THP-1 cells, we performed RT-PCR and Western blotting to determine sirtuin mRNA expression and protein levels after the addition of piceatannol. Our in vitro results showed that piceatannol and its metabolites significantly induced sirtuin mRNA expression and increased sirtuin protein levels, surpassing the effects of resveratrol. To validate these findings in humans, we examined changes in sirtuin mRNA expression in blood after the ingestion of a test food containing 100 mg of piceatannol or a placebo food for two weeks in a double-blind, placebo-controlled, parallel-group study. The results of the analysis of 281 participants confirmed the upregulation of sirtuin mRNA expression due to piceatannol intake. Moreover, stratification by menopausal status revealed that sirtuin mRNA expression was significantly higher as a result of piceatannol intake among postmenopausal women. These findings suggest that piceatannol intake may promote longevity through sirtuin activation, offering potential for longevity therapies and dietary supplements. This study emphasizes the need for further research on piceatannol’s mechanisms of action in the growing field of longevity research.

